# Pulp Fiction Peels Reality: A New Sustainable Platform from Taperebá Coproducts and Green Solvents Modulating Inflammation and Combating Oxidative Stress

**DOI:** 10.1021/acsomega.6c00198

**Published:** 2026-05-29

**Authors:** Leonardo F. A. Chang, Brenda M. Santos, Lívia M. Ferreira, Gabriel R. Farias, Marina A. Radicchi, Eliana F. Gris, Victor C. Mello, Sonia N. Bao

**Affiliations:** † Laboratory of Microscopy and Microanalysis, Department of Cell Biology, Institution of Biological Sciences, University of Brasília, Brasília, Federal District 70910-900, Brazil; ‡ Cooil Cosmetics LTDA, Brasília, Federal District 72622-401, Brazil; § Faculdade de Ciências e Tecnologias em Saúde, University of Brasília, Brasília, Federal District 72220-275, Brazil

## Abstract

This study evaluates a nanostructured lipid carrier (NLC) formulation incorporating taperebá (*Spondias mombin* L.) peel extract and natural deep eutectic solvents (NaDES) as a sustainable antioxidant and anti-inflammatory platform for skin health. The peel extract, rich in polyphenols and carotenoids, was combined with NaDES-based lipids to improve stability and biocompatibility. The NLC-TAP-NaDES demonstrated an average particle size of approximately 150 nm. Human keratinocytes (HaCat) and melanocytes (SH-4) were used to assess cytotoxicity, oxidative stress, and cytokine modulation. NLC-TAP-NaDES showed low toxicity, preserved cell morphology, and significantly reduced superoxide and hydroxyl radicals under hydrogen peroxide stress. The formulation also modulated key cytokines: TNF-α increased under basal conditions, supporting angiogenesis, while IL-6 and IFN-γ were downregulated in keratinocytes, suggesting balanced inflammation and improved barrier function. These findings indicate that NLC-TAP-NaDES provides oxidative protection and modulates inflammatory signaling in skin cells, supporting its potential as a sustainable platform for cosmetic and dermatological applications derived from agricultural byproducts.

## Graphical abstract

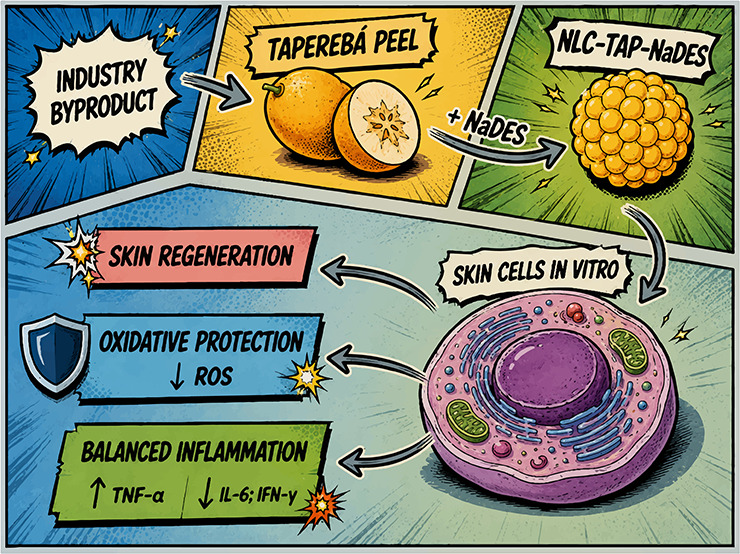

## Introduction

Growing demand for sustainable and eco-friendly technologies is repurposing the production of the next generation of cosmetics and pharmaceuticals, opening space for innovative and green approaches.^1,2^ In regions with strong agricultural activity, large amounts of agricultural biomass are often discarded despite their substantial antioxidant and anti-inflammatory potential, which could be harvested for products with added value.^3^ *Spondias mombin* L., commonly known as Taperebá or Caja, native to the Brazilian Cerrado, is one example. While its pulp is used in culinary traditions, its coproducts, such as the peel, remain underutilized even though they are rich in polyphenols, flavonoids, and carotenoids.^4^

The Cerrado’s biodiversity holds significant potential; however, it remains underexplored due to limited research and challenges in product development, as many of its bioactive compounds exhibit low stability and limited bioavailability. To overcome these challenges involving *S. mombin* compounds, an extract from its peel was incorporated into nanostructured lipid carriers (NLC). These nanoparticles serve as an efficient drug delivery system while offering enhanced stability, improved encapsulation, and controlled release.^5^ In dermatological use, NLC-TAP-NaDES is a promising carrier due to low cytotoxicity, moisture retention from the lipid film, and strong skin penetration, as established in previous NLCs.^5^ These features allow them to coat the epidermis and potentially reach the dermis, improving therapeutic effects.^6^

To enhance biocompatibility and Taperebá’s antioxidant properties, natural deep eutectic solvents (NaDES) were used as part of the lipidic phase of the NLC.^7^ NaDES represent a new class of solvents composed of natural organic compounds such as organic acids, sugars, amino acids, and choline derivatives.^7,8^ When combined in specific molar ratios, these components interact, forming hydrogen bonds and eutectic systems with lower melting points than their individual components.^8^ This unique property enhances their stability, as demonstrated in several recent studies.^7^ Additionally, NaDES are known for their industrial scalability and ecofriendly nature, making them an excellent alternative for incorporation into NLC formulations.^9,10^ Their addition not only boosts the functional properties of the carriers but also aligns with Sustainable Development Goals 9 and 12 proposed by the United Nations, which encourage responsible and innovative production in the pharmaceutical industry and cosmetic applications.^10^

Reactive Oxygen Species (ROS) are free radicals produced by various factors, including ultraviolet light exposure and pollution.^11,12^ The most commonly observed forms of ROS are the superoxide anion (•O_2_−), hydroxyl radical (HO•), and the nonradical hydrogen peroxide (H_2_O_2_).^13^ Elevated levels of these ROS can lead to DNA damage and are related to the pathogenesis of various skin diseases, such as acne, dermatitis, and psoriasis.^13,14^

As the largest organ of the body, the skin is composed of keratinocytes, melanocytes, basal cells, and Langerhans cells and is divided in three layers: the epidermis, dermis, and hypodermis, with the first two being the most affected when skin pathogenesis is present.^15^ This organ acts like a barrier, being exposed to such environmental factors for long periods of time.^15^ Therefore, an active surveillance system when unbalanced, increases proinflammatory cytokines, such as interleukin-6 (IL-6), tumor necrosis factor alpha (TNF-α), and interferon-gamma (IFN-γ), which are widely observed in patients with skin pathogenesis.^16^ They also take part in some physiological processes. TNF-α, for instance, supports extracellular matrix remodeling in keratinocyte cells, which is important for cell regeneration in the inflammatory phase of wound healing.^16,17^

Following the successful development of NLC-TAP-NaDES, a new model of the delivery system was tested by our research group.^18^ This study aims to assess NLC-TAP-NaDES’ effects against ROS and its implications, such as the production of proinflammatory cytokines and morphological alterations in a skin model by assaying its effects on key epidermal cell lines: immortalized keratinocytes (HaCat) and melanocytes (SH-4). This work investigates the formulation’s ability to modulate oxidative stress and inflammatory signaling in epidermal cell models, highlighting a promising strategy for the upcycling of agricultural coproducts into value-added cosmetic and dermatological technologies.

## Results and Discussion

### NLC Profile

The NLC was reformulated and recharacterized according to the protocol previously described by Mello et al. (2025).^18^ Taperebá peels were processed to obtain a phenolic-rich extract. Previous analyses reported for this extract indicate a heterogeneous phenolic composition predominantly containing ellagic acid and quercetin (Mello et al., 2025). Due to the extract’s low aqueous solubility, it was initially prediluted in a choline chloride–citric acid–glycerol NaDES system (1:6, w/v) to ensure homogeneous dispersion prior to incorporation into the lipid phase.

The NLC was subsequently produced by using the phase inversion temperature method. The reformulated system displayed physicochemical characteristics consistent with those previously reported (Table 1).

**Table 1:** Physicochemical Parameters of NLC Formulations: Polydispersity Index (PDI), Hydrodynamic Diameter (HD), and Zeta Potential (ZP)^a^

	PDI	HD (nm)	ZP (mV)
NLC-NaDES	0.26 ± 0.02	196.73 ± 2.37	–2.83 ± 0.27
NLC-TAP	0.23 ± 0.01	199.13 ± 0.91	–4.31 ± 0.16
NLC-TAP-NaDES	0.21 ± 0.03	159.00 ± 0.66	–2.35 ± 0.35

a Adapted from Mello et al. (2025).^18^

Long-term stability assessment demonstrated that NLC-TAP-NaDES maintained its physicochemical integrity for 365 days under refrigerated storage. After this period, the formulation exhibited a mean hydrodynamic diameter of 120 nm, a polydispersity index (PDI) of 0.59, and a zeta potential of −3.43 mV. The relatively higher PDI observed after long-term storage reflects the intrinsic heterogeneity typical of nanostructured lipid carriers, which contain both solid and liquid lipid phases. Such structural complexity often results in broader size distributions when measured by dynamic light scattering, without necessarily compromising the functional stability of the system.

### Cellular Morphological Differences

The study examined the interaction between the cells of the epidermis, the outermost skin layer, and NLC formulated with taperebá coproducts.^18,19^ The epidermis is mainly composed of keratinocytes (about 90%) and melanocytes (about 5%).^20,21^ Melanocytes are responsible for melanin production and help regulate stress responses in nearby keratinocytes.^20^ For this reason, the immortalized human keratinocyte line HaCaT and the human melanocyte line SH-4 were used as in vitro models.

Cell viability was first evaluated with an MTT assay. The results showed that the main formulation components, including NaDES and taperebá extract from its peel, did not increase toxicity. The formulations tested included NLC-NaDES (formulation without taperebá extract, with NaDES), NLC-TAP (formulation with taperebá extract, without NaDES), and NLC-TAP-NaDES (formulation with taperebá extract and NaDES). Nonlinear regression was used to determine the half-maximal inhibitory concentration (IC50) for both cell lines. For HaCat cells, IC50 values were 2.40 μg/mL for NLC-NaDES, 5.22 μg/mL for NLC-TAP, and 7.86 μg/mL for NLC-TAP-NaDES (Figure 1A). For SH-4 cells, IC50 values were 6.54 μg/mL for NLC-NaDES, 5.67 μg/mL for NLC-TAP, and 7.02 μg/mL for NLC-TAP-NaDES (Figure 1B). These findings were consistent with values previously reported by Mello et al. (2025)^18^ in L929 fibroblasts, where NLC-TAP-NaDES showed an IC50 of 5.22 μg/mL. Comparative controls were included to isolate the role of NaDES within the platform, namely NLC-NaDES (vehicle control), NLC-TAP (extract without NaDES), and NLC-TAP-NaDES (extract combined with NaDES). Among the tested systems, NLC-TAP-NaDES consistently exhibited the highest IC50 values in both cell lines, indicating improved cytocompatibility relative to the individual components. This pattern suggests a synergistic interaction between the NaDES system and the polyphenol-rich taperebá extract within the lipid matrix, likely enhancing the dispersion and stabilization of bioactive compounds. Within this same nanocarrier architecture, the mechanistic contribution of NaDES has been previously evidenced through direct comparisons between NLC-TAP and NLC-TAP-NaDES systems, including improvements in thermal behavior and phenolic stabilization under stress conditions.^18^ Taken together, these observations support the role of NaDES as a functional component of the formulation, contributing to the stabilization and effective incorporation of the phenolic fraction within the lipid carrier.

**Figure 1:**
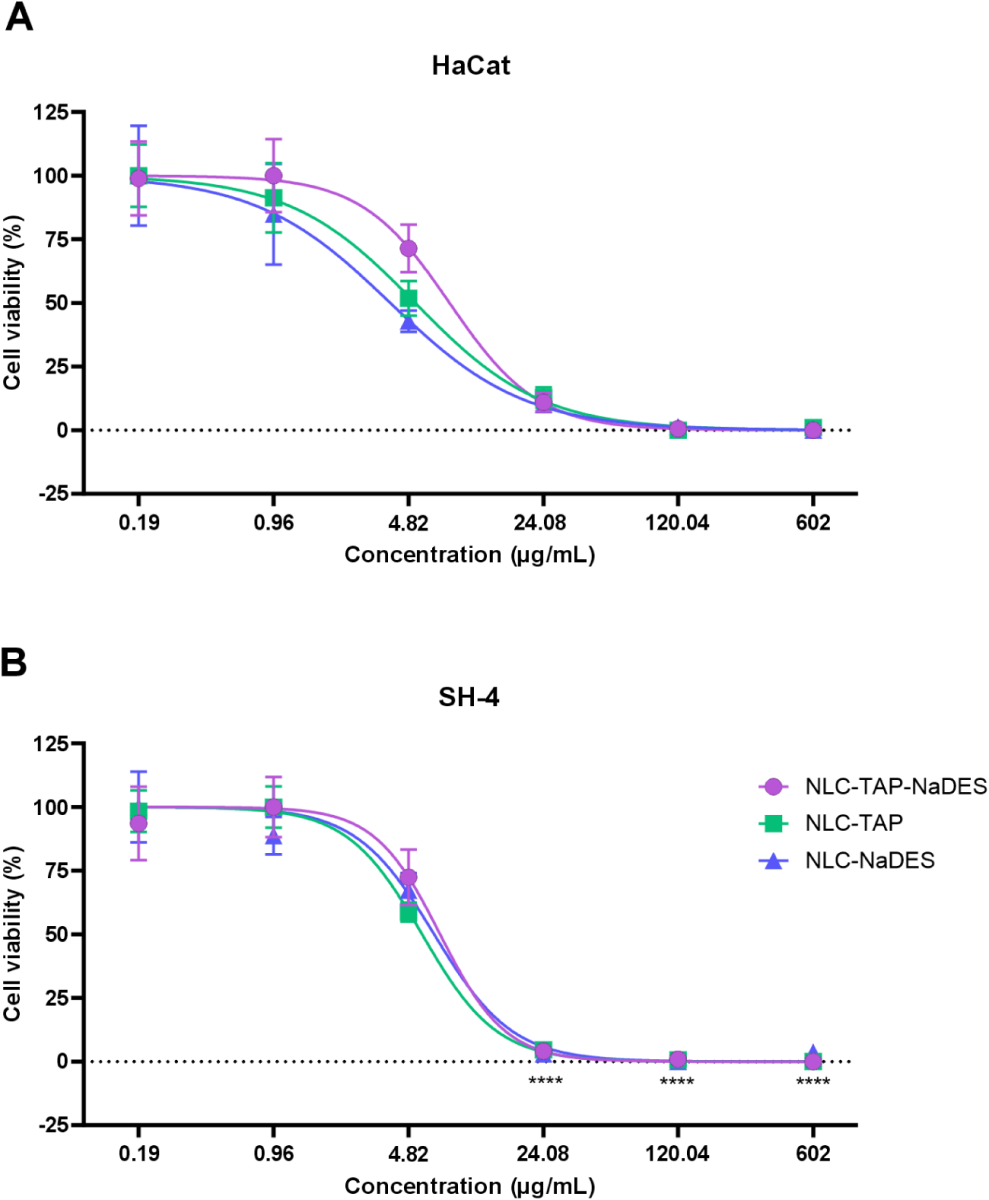
MTT nonlinear dose–response inhibition curve of HaCat (A) and SH-4 cells (B) in the groups NLC-TAP-NaDES, NLC-TAP, and NLC-NaDES.

Based on this, a concentration of 3.61 μg/mL was selected for subsequent assays, representing approximately the IC15 for both lines, where most cells remain viable and subtle formulation effects can be evaluated.

Scanning electron microscopy (SEM) was used to examine the surface morphology. In the HaCaT cell line, the NLC-TAP-NaDES group showed a marked reduction in projections along the cell surface, highlighted by the yellow arrows (Figure 2A-D).^22^ This suggests modulation of melanin delivery to keratinocytes.^20^ The decrease of projections may reflect increased keratin production and organization.^22,23^ As keratinocytes mature, they lose organelles and remodel their cytoskeleton, leading to cell flattening and retraction of projections, which are actin-dependent structures.^22,23^ The cell shifts from an interactive surface to a compact, keratin-filled protective barrier.^24^ These morphological observations are consistent with the cytocompatibility results obtained in the MTT assay, indicating preservation of cellular structure following exposure to the NLC formulations.

**Figure 2:**
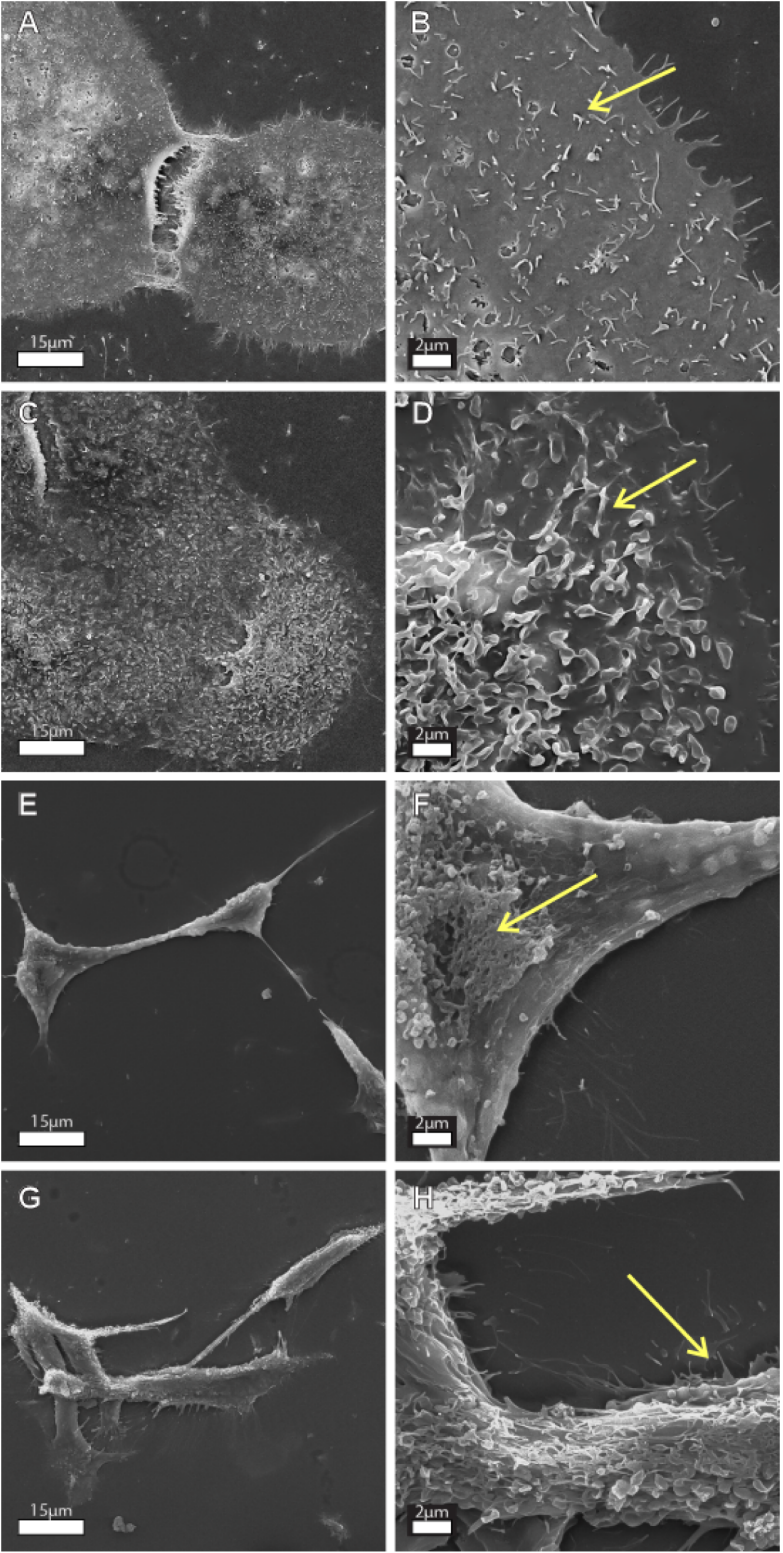
(A,B) HaCat cells treated with NLC-TAP-NaDES (3.61 μg/mL), 1000× and 4500×; (C,D) HaCat cells, control group, 1000× and 4500×, respectively. (E,F) SH-4 cells treated with NLC-TAP-NaDES (3.61 μg/mL), 1000× and 4500×; (G-H) SH-4 cells, control group, 1000× and 4500×, respectively. Yellow arrows mark the control prolonging and projections.

A reduction in melanocyte prolongation was observed in the SH-4 lineage after NLC-TAP-NaDES treatment with the NLC, as highlighted by the yellow arrows. This indicates modulation of cytoskeletal dynamics, likely through the activation of MAPKs and transcription factors, such as AP-1, which regulate cytoskeletal organization, adhesion, and migration (Figure 2E-H).^25^ Dysregulation of these pathways during inflammation affects melanocyte morphology and melanin-related functions.^26,27^ The observed reduction in dendrites supports the hypothesis that NLC-TAP-NaDES modulates these signaling mechanisms, potentially influencing pigmentation and cell communication.

### Reactive Oxygen Species in HaCaT

Previous chemical antioxidant assays performed for this same formulation platform demonstrated significant radical-scavenging activity in DPPH and ABTS systems,^18^ supporting the intrinsic antioxidant potential of the polyphenolic fraction incorporated into the nanocarrier. In the present study, this antioxidant capacity was further investigated at the cellular level. The CellROX Green probe was employed in confocal microscopy to detect intracellular reactive oxygen species, including •OH and O_2_•–, enabling visualization of oxidative stress and evaluation of the effects of NLC-TAP-NaDES treatment in epidermal cell models relevant to skin conditions such as aging, atopic dermatitis, and wound healing.

To further examine the dynamics of oxidative stress modulation, the kinetics of O_2_•– formation were measured using the IVIS Lumina imaging system with lucigenin as a chemiluminescent probe. Luminescence readings were collected at 30 min intervals, allowing real-time monitoring of superoxide production and its modulation by NLC-TAP-NaDES under preventive treatment conditions.

The assays included four groups: the unstimulated group (Uns), with no compound exposure; the positive control (H_2_O_2_), with cells exposed to 500 μM H_2_O_2_ for 1 h; the NLC group (NLC-TAP-NaDES), with cells exposed to the nanocarrier for 24 h; and the preventive group (Preventive), in which cells were pretreated with NLC-TAP-NaDES for 24 h, followed by 500 μM H_2_O_2_ for 1 h. In addition to the cellular assays performed in the present study, the experimental design relies on internal comparisons within the NLC platform to isolate the contribution of each formulation component.

Confocal microscopy analysis (Figure 3A) showed increased fluorescence in the H_2_O_2_ group, indicating elevated ROS levels. In contrast, the NLC-TAP-NaDES group exhibited a fluorescence profile comparable to the unstimulated condition, suggesting that the formulation did not induce oxidative stress. The preventive group displayed reduced fluorescence intensity. Z-stack analysis (Figure 3B-C) further confirmed lower •OH and O_2_•– levels following preventive treatment, as supported by a 37.5% reduction in CellROX Green fluorescence.

**Figure 3:**
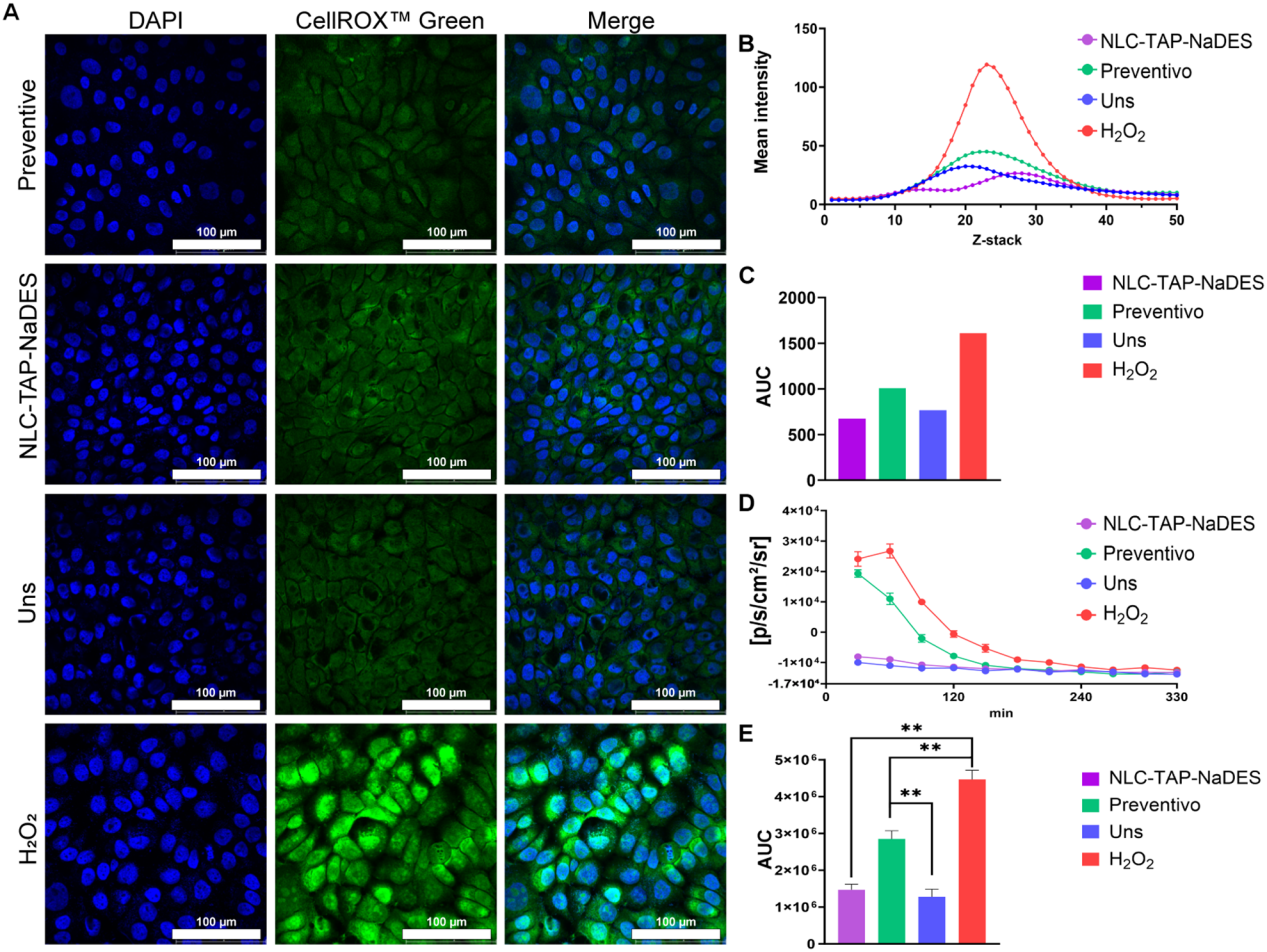
Evaluation of reactive oxygen species in HaCat cells: (A) Confocal microscopy showcasing HaCat cells in the experimental groups of preventive treatment, NLC-TAP-NaDES unstimulated (Uns), and positive control (H_2_O_2_). The cells were stained by DAPI and CellROX Green. (B) Z-stack graph of the quantification of CellROX Green in HaCat cells. (C) Graph of the area under the curve (AUC) of the confocal Z-stack curve (Mean). (D) Curve created by nonlinear regression of the kinetics of superoxide production in HaCat cells in the experimental groups of Uns, H_2_O_2_, NLC-TAP-NaDES, and preventive treatment. (E) Graph of the area under the curve (AUC) of the kinetics of superoxide production curve (Mean ± SEM, *n* = 6) (**P < 0.01).

The antioxidant effects observed in this system are most likely associated with the polyphenolic constituents of the taperebá peel extract, particularly ellagic acid and quercetin, previously identified in this material (Mello et al., 2025). These compounds are known to efficiently scavenge reactive oxygen species through hydrogen atom transfer and single-electron transfer mechanisms. Although carotenoids may also contribute to the antioxidant capacity of *Spondias mombin* fruits, their concentration in peel-derived extracts is typically lower, and their antioxidant activity is more commonly associated with singlet oxygen quenching rather than direct superoxide or hydroxyl radical scavenging. Therefore, the reduction in intracellular ROS observed in the preventive treatment is most plausibly associated with the phenolic fraction incorporated into the NLC system.

Superoxide formation kinetics data (Figure 3D) showed a consistent reduction in O_2_•– production throughout the experiment under preventive treatment. The area under the curve (AUC) showed a 34.14% reduction in total O_2_•– generated in the preventive group compared to the H_2_O_2_ group (Figure 3E). Both graphs, confocal and lucigenin assay, showcased similar ROS profiles, which might indicate that the main ROS generated by the stress due to H_2_O_2_ is O_2_•– in keratinocytes.

Phenolic compounds are essential to the antioxidant capacity of plants, serving as part of their defense system.^28^ Studies on Cerrado fruits have consistently shown high concentrations of bioactive compounds, reinforcing their functional importance in defense systems against intense sunlight, drought, and poor soil.^29^ Previous findings indicate that taperebá peel contains higher total polyphenol levels than its pulp, highlighting its strong antioxidant potential.^4^ This activity arises from the phenolic ring structure, which stabilizes unpaired electrons.^4^ Given its rich bioactive profile, taperebá peel, commonly discarded as waste, represents a valuable resource for food, pharmaceutical, and cosmetic applications, supporting waste reduction and environmental and economic sustainability.

The ROS are essential for many redox reactions and enzyme regulation.^30^ The main ROS include superoxide anion (O_2_•−), hydrogen peroxide (H_2_O_2_), and hydroxyl radical (•OH).^14,30^ At physiological levels, they act as signaling molecules that regulate cell growth, proliferation, differentiation, apoptosis, immune response, and stress adaptation.^14^

ROS are naturally produced in cells, mainly at the inner mitochondrial membrane.^31^ During the electron transport chain, oxygen (O_2_) serves as the final electron acceptor and is normally reduced to water (H_2_O).^14,31^ However, some electrons may prematurely react with O_2_ at complexes I and III, forming superoxide anion (O_2_•−) through a monoelectronic reduction.^32,33^ Under normal conditions, O_2_•– is a weak ROS and is rapidly converted into hydrogen peroxide (H_2_O_2_) by superoxide dismutase (SOD).^14,31,32^ H_2_O_2_ can then be turned into water and oxygen by catalase (CAT) or glutathione peroxidase (GPX), or it can participate in the Fenton reaction with free Fe^2+^, generating the highly reactive hydroxyl radical (•OH) and Fe^3+^.^31–33^ When cells are exposed to excess H_2_O_2_, CAT and GPX become saturated, favoring the Fenton pathway and increasing the production of •OH. High H_2_O_2_ levels also activate NADPH oxidase (NOX) enzymes, which transfer electrons from NADPH to O_2_, generating additional O_2_•–.^14,30,32^ Elevated O_2_•– can also react with nitric oxide (NO) to form peroxynitrite (ONOO−), a highly reactive oxidant^30,31^ (Scheme 1).

**Scheme 1:**
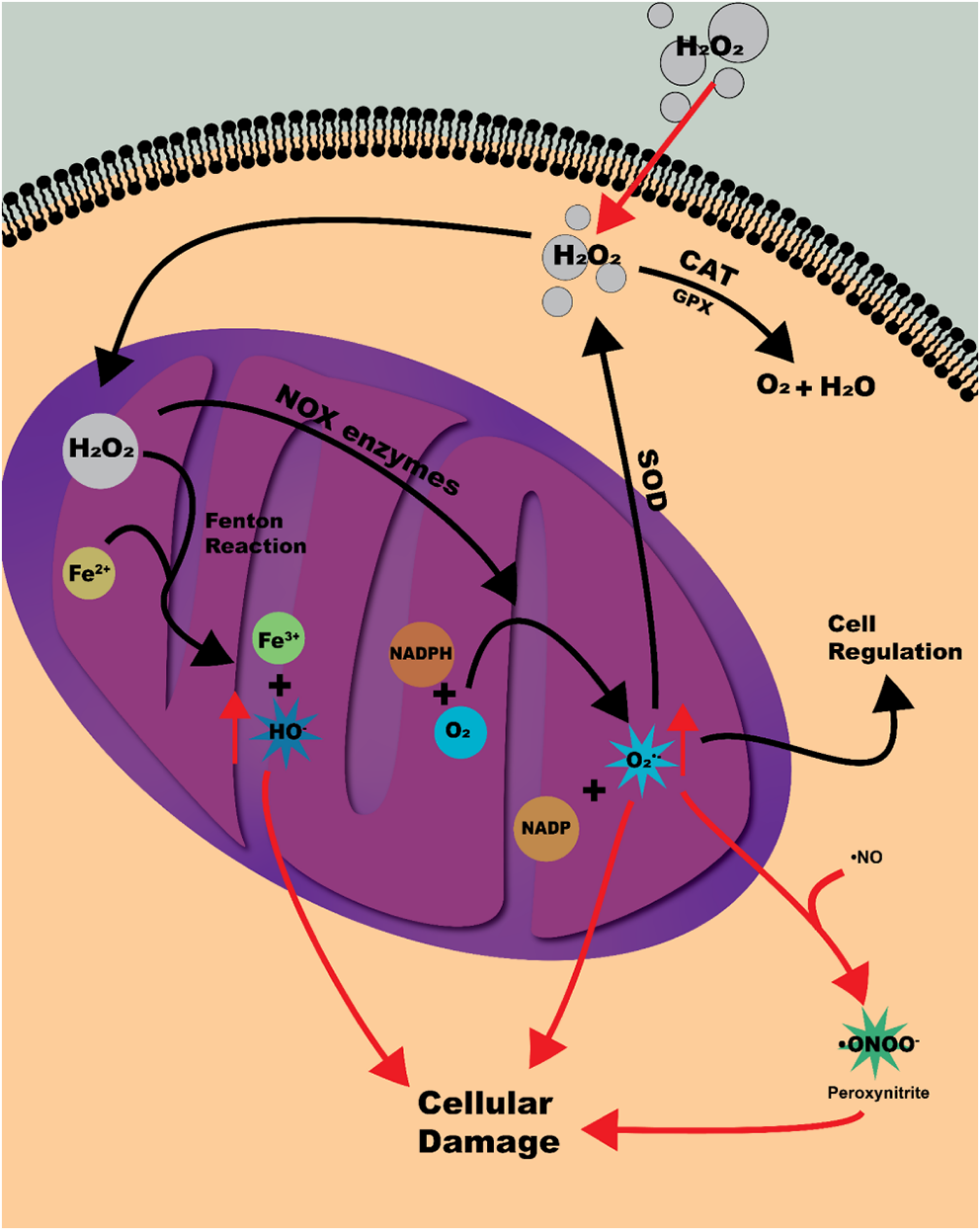
ROS are Mainly Generated in Mitochondria During the Electron Transport Chain^a^

a Leaks at complexes I and III produce O_2_•–, which is converted to H_2_O_2_ by SOD. H_2_O_2_ is detoxified by CAT/GPX, but when it accumulates, the Fenton reaction generates •OH. The increase in O_2_•–, which can form ONOO– with NO. Excess ROS damages DNA, lipids, and proteins, compromising cellular function. Generation of reactive oxygen species when stressed by hydrogen peroxide (H_2_O_2_). Black arrows indicate the regular path of ROS, while red arrows indicate the paths induced upon H_2_O_2_ stimulation.

The hydroxyl radical (•OH) is extremely reactive and attacks DNA bases, deoxyribose sugars, and lipids, initiating lipid peroxidation.^34,35^ Together, O_2_•–, •OH, and H_2_O_2_ can oxidize amino acid residues and cause protein cross-linking, leading to denaturation, loss of enzyme activity, and impaired receptor or transport function.^36,37^ Amino acids containing sulfur, particularly methionine and cysteine, are especially susceptible to oxidation.^38^ Oxidation of methionine’s thioether group often alters protein function, while oxidation of cysteine’s thiol (SH) group is crucial for forming disulfide bonds (S–S), which stabilize tertiary and quaternary protein structures.^38,39^ Excessive ROS can therefore compromise DNA, lipids, and proteins, disrupting cellular function (Scheme 1).^37^

In skin cells, ROS can have both beneficial and harmful effects.^15^ Under physiological conditions, •OH promotes lipid peroxidation, and O_2_•– induces NF-κB dimerization, stimulating keratinocyte proliferation.^31,40^ When ROS levels exceed the normal range, they contribute to pathological processes.^41,42^ In atopic dermatitis, immune inflammation increases O_2_•– and •OH production, worsening the condition.^41^ During aging, catalase activity decreases, leading to H_2_O_2_ accumulation and higher O_2_•– and •OH levels, which activate NF-κB/TNF-α pathways, increase matrix metalloproteinase activity, and accelerate collagen degradation.^42^ In wound healing, excess O_2_•– and iron promote •OH generation through the Fenton reaction, delaying tissue repair.^43,44^

These results support the potential of NLC-TAP-NaDES as a preventive antioxidant platform capable of mitigating oxidative stress responses in skin cell models. It provides preventive protection against environmental damage, aging, atopic dermatitis, and oxidative stress during wound healing.

### Cytokines Quantification

For cytokine quantification, ELISA assays were used to measure TNF-α, IL-6, and IFN-γ. The results showed modulation of TNF-α and IL-6, with TNF-α displaying significant changes in both NLC-TAP-NaDES and preventive treatment groups compared with control groups (unstimulated and H_2_O_2_-stimulated) in HaCat and SH-4 cell lines (Figure 4). These cytokine profiles might be associated with the physicochemical properties of the NLC-TAP-NaDES system, including particle size, lipid composition, and surface characteristics, which can influence cellular uptake and intracellular signaling responses.

**Figure 4:**
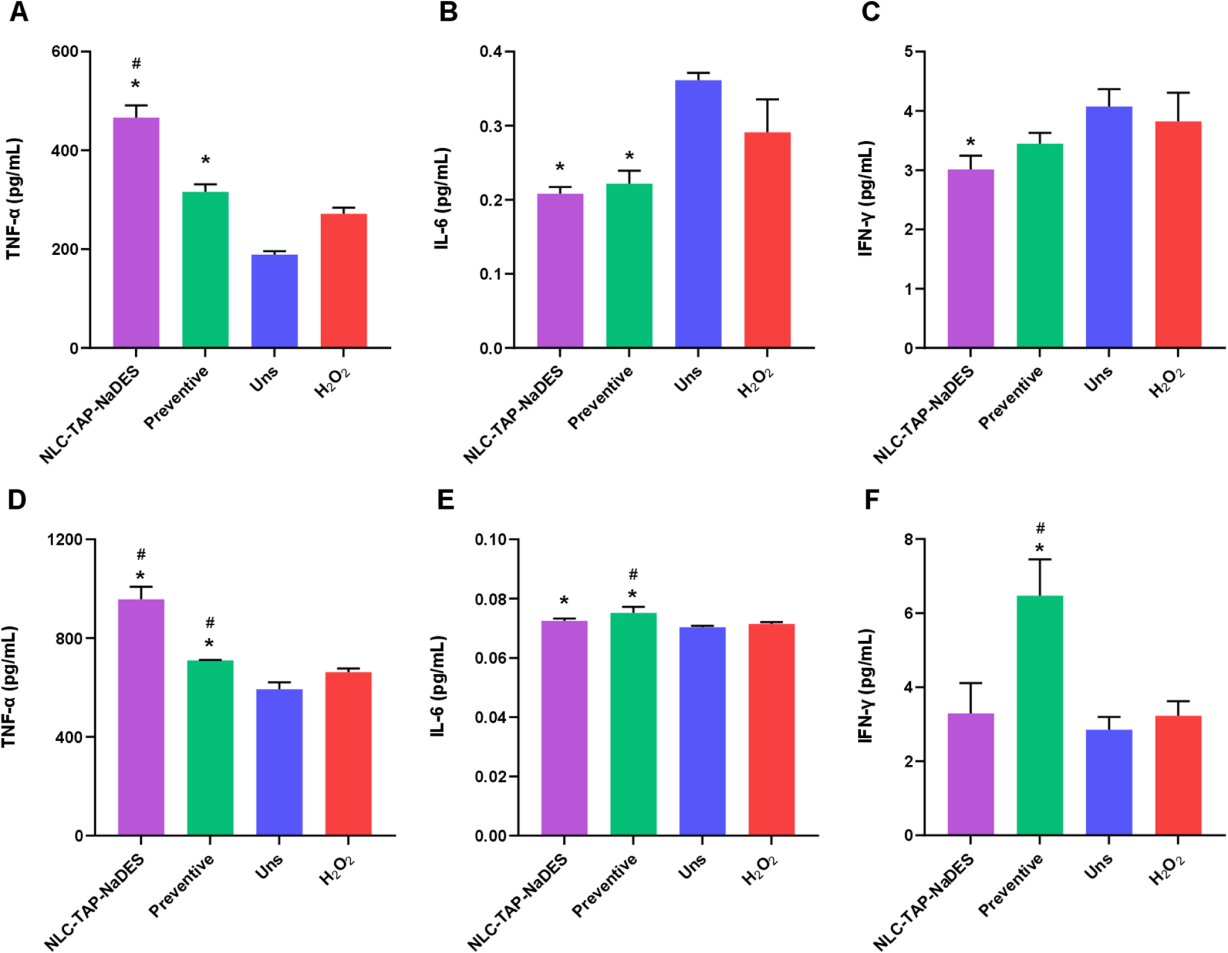
Graph of ELISA results for TNF-α, IL-6, and IFN-γ in HaCat cells (A-C) and SH-4 cells (D-F). The groups are unstimulated, positive control with H_2_O_2_, NLC-TAP-NaDES, and preventive. The data show Mean ± SEM. *P < 0.05 compared with unstimulated. #*P* < 0.05 compared with H_2_O_2_.

TNF-α levels were significantly elevated compared to both control groups. This increase may be partially explained by the nanoscale size of the NLCs, which enhances cellular internalization via endocytic pathways, increasing the intracellular bioavailability of the encapsulated compounds. This may indicate the activation of TNFR1/TNFR2-mediated signaling, leading to the recruitment of adaptor proteins, such as TRADD and TRAF2, and the subsequent activation of the IKK complex, culminating in NF-κB nuclear translocation.^45,46^ In addition, the lipid composition of the NLCs may facilitate membrane interaction and receptor clustering, further promoting signal initiation. Therefore, it plays a crucial role in inflammation by recruiting macrophages and neutrophils and is also critical during the inflammatory phase of wound healing, where NF-κB–dependent transcription can promote the expression of adhesion molecules, chemokines, VEGF, and matrix metalloproteinases (MMPs), thereby stimulating angiogenic signaling.^45–47^ The increase in TNF-α observed in the NLC-TAP-NaDES group, therefore, might suggest the activation of NF-κB–regulated proangiogenic pathways rather than a purely inflammatory escalation.^45,46,48^

During angiogenesis, new capillaries invade the wound clot to establish a microvascular network in the granulation tissue.^48^ This process is initiated by hypoxia-induced stabilization of HIF-1α, which drives VEGF expression and endothelial proliferation, migration, and tube formation.^48^ TNF-α amplifies this response by upregulating VEGF and MMPs, facilitating extracellular matrix remodeling and endothelial sprouting.^48^ In the event of damage, this improved network would supply oxygen and nutrients to the healing area, thereby restoring mitochondrial oxidative phosphorylation and ATP production in proliferating keratinocytes and fibroblasts, supporting cell proliferation and promoting new tissue formation.^49^ Furthermore, it accelerates the recruitment of inflammatory and repair cells, such as fibroblasts, to the wound site through chemokine gradient formation and adhesion molecule expression, thereby enhancing the repair process.^48^

Enhanced angiogenesis also benefits skin appearance.^49^ Age-related decline in angiogenesis is associated with endothelial senescence, reduced VEGF signaling, and diminished nitric oxide bioavailability, leading to impaired microvascular perfusion and extracellular matrix turnover.^50,51^ Stimulating angiogenesis can, therefore, support healthy skin aging by improving microvascular growth, tissue function, and repair efficiency.^51,52^ Better circulation also improves skin appearance, promoting a healthier glow, reduced scarring, and increased skin firmness and thickness.^51^

In the preventive group, TNF-α levels decreased under H_2_O_2_ stress, suggesting attenuation of ROS-mediated hyperactivation of redox-sensitive NF-κB signaling while preserving basal TNF-α activity required for early repair.^53^ This effect may be associated with the antioxidant properties of the lipid matrix and NaDES system, combined with efficient intracellular delivery enabled by the nanoscale structure, which reduces ROS accumulation and downstream signaling overactivation. Excess ROS typically prolongs IKK activation and NF-κB transcriptional activity, leading to sustained cytokine production and tissue damage.^53^ The higher relative TNF-α levels also might suggest that pathways like NF-κB remained active, supporting transcription of genes involved in keratinocyte proliferation and migration at the injury site.^49,54^ Combined with the previously described reduction in ROS, this pattern can speed cell regeneration by facilitating timely resolution of the inflammatory phase and transition to the proliferative phase, lowering infection risk.

IL-6 levels were also modulated by the treatments. In HaCaT cells, IL-6 expression decreased across all experimental groups. This reduction may be linked to the controlled cellular uptake of NLCs, as appropriately sized and biocompatible lipid nanoparticles avoid excessive endosomal stress and prolonged inflammatory signaling. Elevated IL-6 is known to impair wound healing by prolonging inflammation and promoting fibrosis through excessive collagen deposition.^55,56^ At the molecular level, IL-6 signals through the IL-6R/gp130 complex, activating JAK-mediated phosphorylation of STAT3, which drives transcription of antiapoptotic and profibrotic genes.^57^ Persistent STAT3 activation sustains inflammatory signaling and extracellular matrix deposition.^56,57^ The observed reduction indicates improved control of acute inflammation, likely through attenuation of prolonged JAK/STAT3 pathway activation, favoring proper healing and reduced chronic inflammation.^56^

Higher IL-6 activates STAT3 and increases antiapoptotic proteins in melanocytes.^58^ This supports cell survival. IL-6 produced by the melanocytes suggests an autocrine response that helps the cells manage stress in preventive treatment.^58^ Transient STAT3 activation may upregulate cytoprotective genes, such as BCL-2 family members, enhancing resistance to oxidative stress without triggering fibrotic signaling.^59^ The higher IL-6 levels in the preventive group indicate that NLC-TAP-NaDES may enhance survival through an autocrine pathway.

IFN-γ is proinflammatory. It supports antiviral and antimicrobial immunity. It regulates innate and adaptive responses. Nanoparticle surface characteristics are critical in modulating IFN-γ responses, as they influence interactions with cellular receptors and intracellular immune sensors. IFN-γ, with TNF-α, increases monocyte and T-cell recruitment.^60^ IFN-γ signals through JAK1/JAK2, leading to STAT1 phosphorylation and the induction of interferon-stimulated genes (ISGs), including those involved in antigen presentation and antimicrobial defense.^61^ Excessive IFN-γ activity disrupts the skin barrier by altering ceramide composition, likely through STAT1-mediated modulation of lipid metabolism genes, as seen in conditions such as atopic dermatitis.^60–62^ Reduced IFN-γ in keratinocytes in this study may signal restored barrier function and lower chronic inflammation.^63,64^

However, the increase of IFN-γ in melanocytes under preventive conditions may show that NLC-TAP-NaDES improves immune readiness under stress.^63,65^ This selective modulation may result from differences in uptake pathways and intracellular processing, which are influenced by the physicochemical properties. of nanoparticles. Transient STAT1 activation in melanocytes may enhance the expression of antimicrobial peptides and MHC molecules, priming innate immune surveillance without inducing sustained barrier dysfunction.^66^ Controlled IFN-γ expression supports the early inflammatory phase of repair. It recruits immune cells to the injury site and limits microbes and viruses. TNF-α pathways keep keratinocyte proliferation active.^65^ Thus, regulated increases in IFN-γ, coordinated with controlled NF-κB activation, may support balanced inflammatory signaling and efficient progression from inflammation to proliferation.^67^

The NLC-TAP-NaDES treatment demonstrates multifaceted benefits for skin health and wound healing by modulating key cytokines. Overall, the treatment suggests recalibrating redox-sensitive inflammatory networks by differentially modulating NF-κB, JAK/STAT3, and JAK/STAT1 signaling pathways in a cell-type-specific manner. The observed increase in TNF-α in the NLC-TAP-NaDES group might suggest angiogenesis via NF-κB-dependent VEGF and MMP induction, which is crucial for supplying oxygen and nutrients to healing tissues and promoting cell proliferation. The consistent reduction in IL-6 expression in HaCaT cells indicates improved control of acute inflammation and might attenuate the sustained STAT3 signaling and fibrosis-associated transcriptional programs. Finally, the downregulation of IFN-γ in keratinocytes suggests restored barrier function and reduced chronic inflammation, while the controlled increase in IFN-γ in melanocytes under preventive conditions suggests enhanced immune priming. Collectively, these synchronized signaling modifications establish a mechanistic foundation for the preventive therapeutic efficacy of NLC-TAP-NaDES. These results emphasize that the optimization of NLC physicochemical properties is critical not only for achieving delivery efficiency but also for governing downstream cytokine activity and overall therapeutic results.

## Conclusion

By integrating taperebá (*Spondias mombin* L.) peel extract into NaDES-enabled nanostructured lipid carriers, we demonstrate a sustainability-anchored platform that attenuates oxidative burden while recalibrating inflammatory tone in keratinocyte cells. Across keratinocytes and melanocytes, the formulation lowered ROS output and downmodulated IL-6 and IFN-γ, yet preserved prorepair TNF-α cues, collectively indicating modulation of redox balance and inflammatory signaling pathways associated with skin homeostasis, with low cytotoxicity in epidermal cell models. Beyond performance, the work establishes a translational route to value Cerrado’s coproducts, an underused biogenomic hub, into safe, efficacious cosmeceuticals framed by access-and-benefit-sharing compliance, fair-trade sourcing, and auditable traceability. This coupling of green chemistry, circular feedstocks, and nanoenabled delivery promotes Sustainable Development Goals by aligning innovation with responsible production and local value capture. Future studies should further investigate the encapsulation efficiency and release behavior of the polyphenolic compounds present in the taperebá peel extract in order to optimize the technological performance of this nanocarrier platform. As such, NLC-TAP-NaDES is positioned not merely as a single formulation but as an extensible design logic for upcycling agrobiodiversity into dermatological technologies that reconcile efficacy, equity, and ecosystem stewardship.

## Materials and Methods

### Formulation of the Nanostructured Lipid Carrier

The NLC containing Taperebá byproduct extract was prepared and characterized following Mello et al. (2025).^18^ The formulation used Murumuru butter and Buriti oil (Amazon Oil) as lipid components, Brij O10 as a surfactant, and the NaDES was composed of choline chloride, glycerol, and citric acid.

Ripe taperebá fruits (*Spondias mombin* L.) were collected by Embrapa Cerrados (CPAC) in Planaltina, Federal District, Brazil (15°54′16.50″ S, 47°22′37.49″ W, 855 m altitude), an area within the Cerrado biome. The project was registered in the National Genetic Heritage and Associated Traditional Knowledge Management System (SisGen) under codes A002D4B and A235193. The fruit peels were manually separated, dried at room temperature for 24 h, protected from light, and the extract was obtained as described by de Brito et al. (2022).^4^

The extract was dissolved in the NaDES and incorporated into the lipid phase. The NLCs were produced by hot emulsification, with the lipid and aqueous phases heated separately to 80 °C for homogeneity. The phases were then combined under high-speed stirring to form an emulsion, followed by rapid cooling in an ice bath to solidify the lipid matrix and stabilize the nanoparticles.

### Cell Maintenance

Human keratinocyte cells (HaCat) and human melanocyte cells (SH-4) were cultured in Dulbecco’s Modified Eagle Medium (DMEM), supplemented with 10% (v:v) FBS and 1% (v:v) antibiotic solution. The cells were maintained in a humidified incubator with 5% CO_2_ at 37 °C.

### Cytotoxicity of Human Keratinocytes (HaCaT) and Human Melanocytes (SH-4)

The 3-(4,5-dimethylthiazol-2-yl)-2,5-diphenyltetrazolium bromide (MTT) assay was conducted to evaluate cell viability in the control group and experimental groups: NLC-NaDES, NLC-TAP, and NLC-TAP-NaDES. The tested concentrations were 602 μg/mL, 120.04 μg/mL, 24.08 μg/mL, 4.82 μg/mL, 0.96 μg/mL, and 0.19 μg/mL. In 96-well plates, 5 × 10^3^ human keratinocytes (HaCat) were seeded per well, while in other plates, 5 × 10^3^ human melanocytes (SH-4) were seeded per well, for a 24-h incubation period to ensure adhesion. Subsequently, the cells were exposed to different concentrations of NLC. After 24 h of treatment, the wells were incubated for 4 h in the dark at 37 °C with MTT solution at 0.5 mg/mL. After incubation, the MTT solution was replaced with DMSO per well to dissolve the formazan crystals. Plate readings were performed in absorbance mode at 595 nm. The assay was conducted in quadruplicate wells for each concentration in three independent experiments.

### Cell Line Analysis in Scanning Electron Microscopy (SEM)

To visualize potential changes in the shape and surface of cell lines after 24 h treatments at a concentration of 3.61 μg/mL, the cells were analyzed using scanning electron microscopy (SEM). Atotal og 5 × 10^5^ cells were seeded on 18 × 18 mm coverslips. After cell adhesion, they were either treated or not treated for 24 h with NLC-TAP-NaDES at a concentration of 3.61 μg/mL.

Subsequently, the culture medium was discarded, and the cells were washed twice with 1× PBS. Then, the cells were fixed with Karnovsky’s fixative solution (containing 2% glutaraldehyde, 2% paraformaldehyde, and 3% sucrose in 0.1 M sodium cacodylate buffer, pH 7.2) and left to rest overnight. The next day, the fixative was removed, and the cells were washed with 0.1 M sodium cacodylate buffer (pH 7.2).

The coverslips were then incubated in 2% osmium tetroxide vapor for 30 min and, after this period, washed with distilled water. Dehydration was performed in an increasing series of acetone (50% to 100%), followed by critical point drying (Balzers, CPD 030, Germany). Finally, the samples were metallized using an SCD 500 Metallizer (LEICA, Germany) and analyzed using a scanning electron microscope operated at an acceleration voltage of 15 kV using a JEOL JSM 7001 F (Tokyo, Japan).

### Confocal ROS Evaluation

HaCat cells were seeded onto circular coverslips at 3 × 10^4^ cells per well and treated according to each experimental group: Unstimulated (Uns), H_2_O_2_-treated (500 μM), NLC-TAP-NaDES (3.61 μg/mL for 24 h), or preventive treatment (NLC-TAP-NaDES for 24 h followed by 500 μM H_2_O_2_). After treatments, cells were stained with 5 μM CellROX Green and 500 nM DAPI, and analyzed using a Leica TCS SP5 confocal microscope (Leica Microsystems, Wetzlar, Germany). Z-stacks were acquired with 50 steps, using the 488 nm laser for CellROX Green and the 405 nm laser for DAPI.

### Kinetics of Superoxide Formation (O_2_^–^)

The kinetics of superoxide production assay were carried out by seeding 5 × 10^4^ HaCat cells per well in a 24-well plate, followed by the addition of 10% (v:v) lucigenin to each well. The experiment was structured into three groups: Unstimulated (Uns), H_2_O_2_ 1 mM treated (serving as the positive control), NLC-TAP-NaDES treatment with 3.61 μg/mL of the NLC, and preventive treatment (NLC-TAP-NaDES exposure for 24 h at a concentration of 3.61 μg/mL, succeeded by H_2_O_2_ 1 mM). Chemiluminescence was measured using an IVIS LUMINA III (Revvity Inc., Waltham, Massachusetts, United States) instrument with an open emission filter, performing 5 min exposures with a binning of 4 in the D field of view, over a span of 11 readings, with a 25 min delay between each analysis. A circular region of interest (ROI) was defined for each well. Chemiluminescence intensity was measured as mean radiance (photons/s/cm^2^/sr) and corrected by subtracting the blank without lucigenin. Radiance–time curves were generated in GraphPad Prism 10. The software calculated the area under the curve using Riemann sums to compare total oxidative load, as shown in the equation below:

$$\mathrm{AUC total}=\overset{n-1}{\underset{i=1}{\sum }}\frac{\left(I\left(i\right)-\mathrm{Min}\mathrm{Value}\right)+\left(I\left(i+1\right)-\mathrm{MinValue}\right)}{2}\times \frac{t\left(i+1\right)-t\left(i\right)}{n}$$

*I*: intensity (photons/s/cm^2^/sr); *t*: time (min); *i*: interval; MinValue: minimal value.

### Cytokines Profile

For the cytokine quantification, an ELISA assay was used. A total of 2 × 10^4^ cells were seeded in a 24-well plate for each cell line. After adhesion, they were treated according to each group.

The groups were designated as unstimulated (Uns), H_2_O_2_-treated (500 μM), NLC-TAP-NaDES (3.61 μg/mL for 24 h), and Preventive treatment (NLC-TAP-NaDES for 24 h followed by 500 μM H_2_O_2_ exposure).

After each treatment, supernatants were collected to map cytokine production stimulated by the therapy using an ELISA kit. Cytokines TNF-α, IFN-γ, and IL-6 were assayed by using commercial kits and according to the protocols provided by the manufacturer. Absorbance values were generated from readings on a spectrophotometer, Varioskan LUX (Thermo Fisher Scientific, Massachusetts, United States), at a wavelength of 450 nm. The determination of cytokine concentrations was performed using specific standard curves for each cytokine, presented in absolute values in picograms per milliliter (pg/mL).

## Statistical Analysis

For the cell viability analysis, absorbance values were plotted in an XY table, log-transformed (Log_10_), normalized, and fitted with a nonlinear dose–response inhibition curve using GraphPad Prism version 10.

For the parametric tests, it used the Student *t*-test for comparing two groups or the ANOVA test for three or more groups. Statistical analyses for nonparametric tests used the Mann–Whitney U test for comparing two groups or the Kruskal–Wallis test for three or more groups, using GraphPad Prism version 10.

## Data Availability

The data supporting the findings of this study are available within the article itself. This study is a direct continuation of our previous work entitled “Advanced Solubilization of Brazilian Cerrado Byproduct Extracts Using Green Nanostructured Lipid Carriers and NaDESs for Enhanced Antioxidant Potentials; doi: 10.3390/antiox14030290. Any additional raw data generated or analyzed during the present study are available from the corresponding author upon reasonable request.
